# Effects of soil treated fungicide fluopimomide on tomato (*Solanum lycopersicum* L.) disease control and plant growth

**DOI:** 10.1515/biol-2022-0069

**Published:** 2022-07-25

**Authors:** Lili Jiang, Hongyan Wang, Xiaojuan Zong, Xiaofang Wang, Chong Wu

**Affiliations:** Shandong Institute of Pomology, Shandong Academy of Agricultural Science, 66 Longtan Road, Tai’an 271000, Shandong, P. R. China; Department of Plant Protection, Shandong Agricultural University, Tai’an 271018, Shandong, P. R. China

**Keywords:** fluopimomide, tomato, soil-borne disease, soil enzyme, micro biomass

## Abstract

Fluopimomide is a novel acid amide fungicide registered for the control of many plant pathogens. In the present study, the effects of soil-treated fluopimomide on soil micro biomass, disease incidence, plant growth, soil enzyme activity, and marketable yield of tomato (*Solanum lycopersicum* L.) were investigated via field trial. In addition, the application prospect in China was also evaluated. In the experiment, five treatments with three replications and a randomized complete block design were followed. The treatments were: furrow application of fluopimomide (25% suspension concentrate, SC) at the dosage of 375, 750, and 1,500 g ha^−1^, which was recommended, double recommended, and quadruple recommended dosages, respectively. Besides, common control fungicide fluopicolide (5% SC) furrow was applied at recommended application dosages of 750 mL ha^−1^, and a non-treated control was also undertaken. Results indicated that fluopimomide exhibited no effects on the amount of soil bacteria and actinomycetes, and its inhibition effect on fungi amount could be recovered at 60 days after treatment (DAT). With the recommended application dosage, fluopimomide could efficiently reduce the number of plant pathogens in soil by 79.56–85.80%, significantly reduce the disease incidences in tomato plants by 80.00–88.24%, and improve plant height by 13.25–24.05% and marketable yield by 16.88%. Furthermore, soil enzymes exhibited a complex response to fluopimomide, and AOB and *nifH* gene copy numbers were increased by the double and quadruple recommended dosage of fluopimomide. Based on the above results, fluopimomide could be recommended as an efficient fungicide for the tomato field.

## 1 Introduction

Tomato (*Solanum lycopersicum* L.) is an important vegetable crop [1]. In China, the annual tomato production reached 50 million MT. However, due to long-term continuous cropping and mismanagement, tomato diseases have been increasing over time. The most common diseases are: wilt, blight, and gray mold, which are caused by *Fusarium oxysporum*, *Phytophthora* spp., and *Botrytis cinerea*, respectively [2,3,4]. Yield loss caused by above diseases reached 10–30% in general plots, and 50% in serious plots. Fungicide application is usually one of the main components in tomato production.

Fluopicolide effectively suppressed sporangium formation, zoospore germination, and mycelial growth of Phytophthora pathogen [5], and could significantly reduce the incidence of watermelon fruit rotting in the Carolinas [6]. In China, its preparation named Yinfali Suspension has been widely used for blight control of pepper (*Capsicum frutescence* L.), potato (*Solanum tuberosum* L.), tomato (*S. lycopersicum*), cucumber (*Cucumis sativus* L.), and other vegetables, its joint application with bio-fungicides could control cucumber diseases more efficiently.

**Figure 1:**
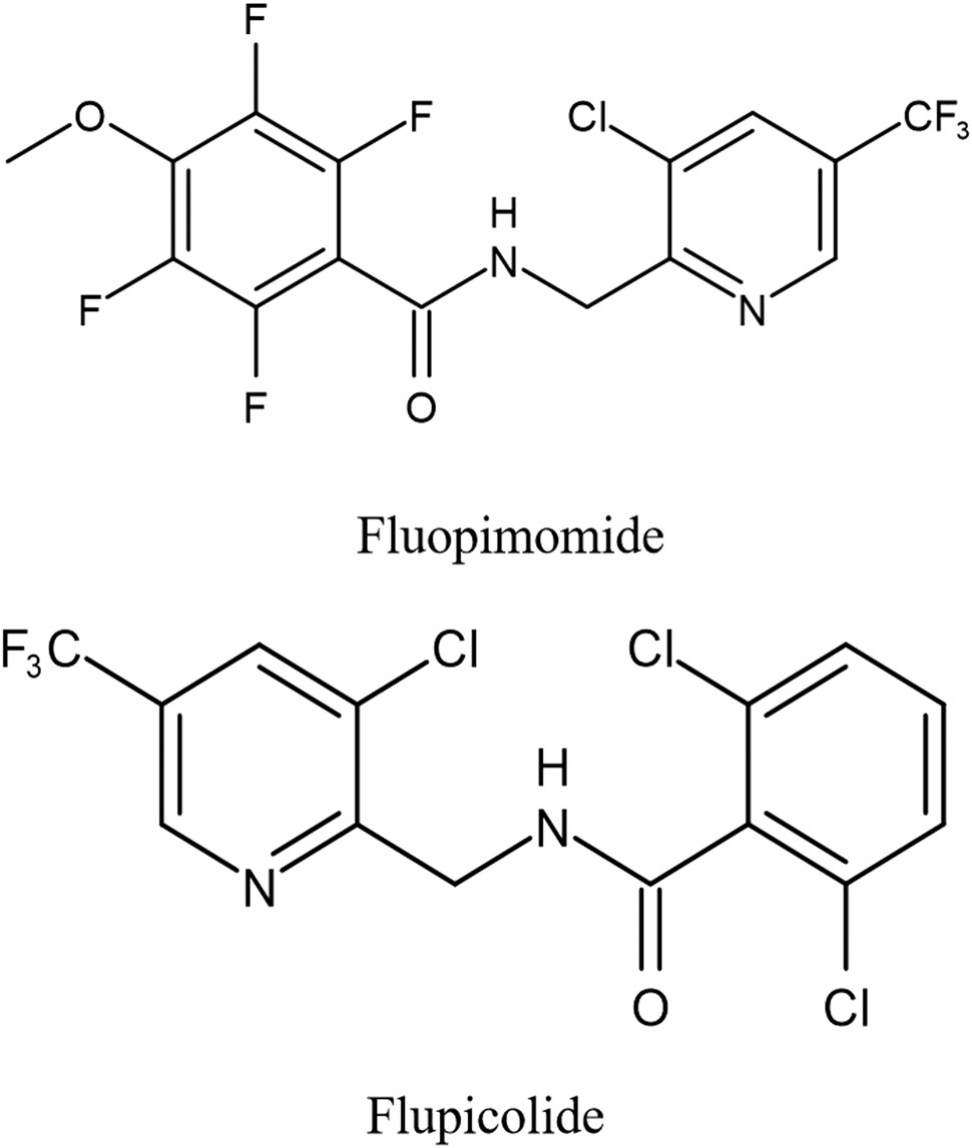
Structural formula of fluopimomide and fluopicolide.

Fluopimomide is a new fluorinated benzamide fungicide developed by Shandong United Pesticide Industry Co. Ltd, China in 2010, it has a similar structure to fluopicolide [7] (Figure 1). Its chemical name is *N*-(3-chloro-5-trifluoromethyl-pyridine-2-methyl-2,3,5,6-tetrafluoroethane-4-methoxy-benzamide) [8]. In China, fluopimomide has been reported to be efficient for *Phytophthora* and nematode control [9]. Taking into account the structural similarity of the two compounds, it is recognized that fluopimomide would be effective in preventing oomycetes and other vegetable diseases. However, with the addition of 4 fluorine atoms and a methoxy group in fluopimomide, there might be a great difference between fluopicolide and fluopimomide in sterilization virulence, bactericidal spectrum, mode of action, and mechanism of action, etc.

In the previous study, bactericidal spectrum of the innovative fungicide fluopimomide was measured by *in vitro* bioassays, and exhibited EC_50_ values of 0.97, 2.36, and 3.59 μg mL^−1^ for *B. cinerea*, *Phytophthora*, and *F. oxysporum*, respectively [10]. However, little information is available about its application effects on the tomato field. The aims of the present study were: (a) to determine the control efficacy of fluopimomide on detrimental pathogens in tomato field, including *F. oxysporum*, *Phytophthora,* and *B. cinerea*, (b) to investigate its micro-ecology effect on tomato planted soil, and (c) to identify the influence of fluopimomide on tomato plant growth and marketable yield.

## 2 Materials and methods

### 2.1 Chemicals and reagents

Fluopimomide (purity = 98%) and fluopicolide (purity = 96%) were both provided by Shandong United Pesticide Industry Co. Ltd, China.

### 2.2 Field experiment design

Field experiments were arranged in the autumn cropping seasons of 2018–2019 and 2019–2020 in a commercial greenhouse near Fang county, Tai’an, China (N35°58′13″, E117°12′13″). Six-week-old “Jinpeng” tomato seedlings were transplanted on August 23, 2018 and August 18, 2019.

The soil physicochemical properties of the experimental sites were: sand 29.17%, silt 70.49%, clay 0.34%, organic matter 18.95 g kg^−1^ soil, and pH 7.1. This site suffered heavily from soil-borne diseases, such as *F. oxysporum*, *Phytophthora,* and *B. cinerea*. In conventional farm operations, compound fertilizer of 15N–15P_2_O_5_–15K_2_O was broadcast applied at the dosage of 750 kg ha^−1^ as base.

Five treatments with 3 replicates in each were arranged in a random block design, where 25 seedlings were planted per plot (7.20 m × 0.75 m). The treatment programs were: (a) laboratory-made fluopimomide (25% SC, a.i.) furrow-application was done at a dosage of 375 g ha^−1^, it is the field recommended application dose; (b) fluopimomide (25% SC, a.i.) furrow-application dose of 750 g ha^−1^; (c) fluopimomide (25% SC, a.i.) furrow-application dose of 1,500 g ha^−1^; (d) laboratory-made fluopicolide (5% SC, a.i.) furrow-application dose of 750 mL ha^−1^, it is the field recommended dose; and (e) non-treated control. Evaluation of 2 and 4 folds of recommended application dosage is necessary for novel pesticide crop safety verification.

### 2.3 Sampling

20, 40, and 60 DAT, a soil column cylinder with a diameter of 5 cm was used to sample soil nearby tomato plants at a depth of 0–10 cm, and random 20 points were included for each plot. Each collected soil sample (0.5–1.0 kg) was sieved (1 mm mesh) and separated into two parts. Part one was stored at 4°C for the microbiological and enzyme activities test, and the test was completed within less than a week. Part two was treated with liquid nitrogen and stored at −80°C for DNA extraction.

### 2.4 Enumeration of microbial population

The amounts of fungi, bacteria, and actinomycetes were evaluated using the serial ten-fold dilution (10-2–10-7) method [11], where 45 mL of sterile water mixed with 5 g soil was regarded as 10^−1^ dilution. Fungi, including yeast, were counted on Martin’s medium, with pH 6 and containing 150 mg L^−1^ streptomycin. Bacteria were counted on a selective medium prepared with glucose 1 g L^−1^, proteose peptone 3 g L^−1^, yeast extract 1 g L^−1^, K_2_PO_4_ 1 g L^−1^, agar 15 g L^−1^, and cycloheximide 100 mg L^−1^. Actinomycetes were counted on improved GAO No.1 medium with pH 7.4–7.6 [12]. The number of *F. oxysporum* in soil was counted on the PCNB selective medium containing KH_2_PO_4_ 1.0 g L^−1^, MgSO_4_·7H_2_O 0.5 g L^−1^, peptone 5.0 g L^−1^, agar 20.0 g L^−1^, with streptomycin 0.30 g L^−1^ and 75% quintozene wettable powder 1.0 g L^−1^ added before usage [13]. The amount of *Phytophthora* was measured in a PDA medium with 3-hydroxy-5-methylisoxazole, benomyl, nyastatin, pentachloronitrobenzen, rifampicin, and ampicillin added at the concentration of 100, 10, 25, 25, 10, and 50 mg L^−1^, respectively [14]. The *B. cinerea* selective medium included basic components (NaNO_3_ 1.0 g L^−1^, KCl 0.15 g L^−1^, K_2_HPO_4_ 1.2 g L^−1^, MgSO_4_·7H_2_O 0.5 g L^−1^, glucose 20.0 g L^−1^, agar 25.0 g L^−1^) as well as fungicide components (pentachloronitrobenzen 0.012 g L^−1^, penicillin 0.05 g L^−1^, chloramphenicol 0.05 g L^−1^, sulphate streptomycin 0.05 g L^−1^, CuSO_4_ 2.2 g L^−1^, and Rubigan 0.01 mL L^−1^) added after sterilization [15]. Dilution ratios were chosen properly according to their present amounts in the soil. From the diluted solution, 100 μL was smeared on the various medium plates, each with five replications. After cultivation for 5 days at 25°C, the plate with colony amounts between 10 and 100 was used for the eventual calculation of microbe numbers and population densities. The data were reported as cfu g^−1^.

### 2.5 Disease assessment in plants

After the transplant, incidences of wilt, blight, and gray mold of ten random plants in each plot were recorded at intervals of 20, 40, and 60 days. Levels of disease severity were assessed by visually estimating the percentage of diseased surface, and graded as: 0 = 0%, 1 = 1–9%, 2 = 10–24%, 3 = 25–49%, 4 = >50% of surface affected. Wilt was calculated by spot on stem base, while blight and gray mold were weighted by round and angle spots on leaves, respectively. The disease index was calculated using the following formula:

\text{Disease}\hspace{.5em}\text{index}=\frac{\sum \text{(The}\hspace{.5em}\text{relative}\hspace{.5em}\text{score}\times \text{Plant}\hspace{.5em}\text{numbers}\hspace{.5em}\text{in}\hspace{.5em}\text{the}\hspace{.5em}\text{score)}}{\text{Total}\hspace{.5em}\text{plant}\hspace{.5em}\text{numbers}\times \text{4}}\times \text{100,}]

### 2.6 Assays of soil enzymatic activities

Analysis of soil dehydrogenase and urease activity was done according to the methods of Lebrun et al. [16]. Soil phosphatase activity was tested using the method of Wang et al. [17]. The invertase activity was measured following the modified method of Ohshima et al. [18].

### 2.7 Quantitative PCR analysis of soil nitrogen-related genes

The qPCR analysis of AOA, AOB, *nif*H, and *nir*S was performed with ABI 7500 Real-Time PCR System and 7500 System Software-SDS 2.2 by absolute quantification method, and the primer information is shown in Table 1. The reaction system consisted of 2 × SuperReal PreMix 10 μL, primers 0.3 μmol L^−1^, and cDNA template 2.5 μL. The procedure of qPCR was performed at 95°C for 15 min and followed by 40 cycles of 95°C for 10 s, annealing (53°C for AOA, 56°C for AOB, 58°C for *nif*H and *nir*S) for 20 s, and 72°C for 1 min.

**Table 1:** Primer sequence

Gene	Primer sequence
AOA	L: 5′-STAATGGTCTGGCTTAGACG-3′
	R: 5′-GCGGCCATCCATCTGTATGT-3′
AOB	L: 5′-GGGGTTTCTACTGGTGGT-3′
	R: 5′-CCCCTCKGSAAAGCCTTCTTC-3′
*nif*H	L: 5′-AAAGGYGGWATCGGYAARTCCACCAC-3′
	R: 5′-TTGTTSGCSGCRTACATSGCCATCAT-3′
*nir*S	L: 5′-CCTAYTGGCCGCCRCART-3′
	R: 5′-CGTTGAACTTRCCGGT-3′

### 2.8 Influence of fluopimomide on tomato plants

20, 40, and 60 DAT, the plant height of randomly chosen ten plants in each plot was measured. Fruits were harvested twice (85 and 120 DAT) when they were mature, and the marketable yield was calculated.

### 2.9 Statistical analysis

As there were no significant differences between experiments over the 2 years, data from the two experiments were combined for analysis and interpretation. The data were analyzed statistically by the Duncan test with a significance level of *p* < 0.05.

## 3 Results and discussion

### 3.1 Effect of fluopimomide on the abundance of fungi, bacteria, and actinomycetes

Soil microbes are basic components of soil ecology and are highly sensitive to environmental changes [19]. The diversity and abundance of soil microbial communities are the important indices for gaining knowledge of modern soil microbiology. Still, it is the main indicator of risk assessment for pesticide application in agricultural fields [20]. So, in the present study, the application of novel fungicide fluopimomide required tests to determine the effect on the soil environment. As shown in Table 2, under the concentration of 1, 2, and 4 folds of field recommended dosage, fungicide fluopimomide reduced the amounts of fungi significantly (*p* < 0.05), while it showed no influence on bacteria and actinomycetes. This result falls following Ji et al., who had reported the excellent compatibility of fluopimomide with *Bacillus*. 20, 40, and 60 DAT, the amounts of fungi treated by fluopimomide increased gradually with the time-lapse and recovered to the control level at 60 DAT under the recommended dosages. However, the treatments with 2- and 4-fold still inhibited the amount of fungal population. The control fungicide fluopicolide exhibited similar effects with fluopimomide on the amounts of fungi, bacteria, and actinomycetes. Overall, fluopimomide had no significant negative effects on soil bacteria and actinomycetes, and the effects on fungal populations could be recovered within 60 days, indicating a less negative effect on soil microbe.

**Table 2:** Effect of fluopimomide on amounts of the bacteria, fungi and actinomycetes in tomato field

Treatment	20 days	40 days		60 days
**Bacteria/(×10** ^**6**^ ** cfu g** ^**−1**^)				
Fluopimomide 375	1.72 ± 0.13a^†^		1.95 ± 0.38a	1.30 ± 0.20a
Fluopimomide 750	1.68 ± 0.26a		1.75 ± 0.35a	1.35 ± 0.18a
Fluopimomide 1,500	1.70 ± 0.24a		1.68 ± 0.26a	1.42 ± 0.25a
Fluopicolide 750	1.60 ± 0.24a		2.12 ± 0.42a	1.30 ± 0.25a
Control	2.02 ± 0.20a		2.22 ± 0.24a	1.65 ± 0.17a
**Fungi/(×10** ^**3**^ ** cfu g** ^**−1**^)				
Fluopimomide 375	2.80 ± 0.38bc		3.55 ± 0.26b	3.58 ± 0.25ab
Fluopimomide 750	2.65 ± 0.26bc		3.68 ± 0.17b	3.25 ± 0.35bc
Fluopimomide 1,500	2.30 ± 0.31c		3.30 ± 0.21b	3.15 ± 0.15bc
Fluopicolide 750	3.15 ± 0.10ab		3.72 ± 0.46b	4.02 ± 0.35ab
Control	3.52 ± 0.45a		5.05 ± 0.14a	4.32 ± 0.17a
**Actinomycetes/(×10** ^**4**^ ** cfu g** ^**−1**^)				
Fluopimomide 375	2.20 ± 0.20a		2.78 ± 0.22a	3.15 ± 0.22a
Fluopimomide 750	1.92 ± 0.24a		2.40 ± 0.42a	2.98 ± 0.38a
Fluopimomide 1,500	1.75 ± 0.23a		2.52 ± 0.34a	3.12 ± 0.29a
Fluopicolide 750	1.85 ± 0.23a		2.60 ± 0.45a	3.08 ± 0.42a
Control	2.48 ± 0.25a		3.10 ± 0.54a	3.60 ± 0.46a

† The results showed as mean value ± std. error, different letters in each column showed the significant difference at 0.05 level, and the same as follows.

### 3.2 Effects of fungicide fluopimomide on the amount of soil-borne pathogens

As a greenhouse vegetable, disease management is one of the most essential components of tomato production. The primary soil-borne diseases, wilt (*F. oxysporum*), blight (*P. infestans*), and grey mold (*B. cinerea*), have caused severe yield loss throughout the world [21], and current systematic fungicides are efficient for these disease control [22,23]. Manikandan et al. reported that the high *Fusarium* gene level in tomato planted soil suffered from wilt [24]. In our assay, the soil application of fungicide fluopimomide has reduced the amounts of three typical soil-borne pathogens, especially *B. cinerea* and *F. oxysporum* (the inhibition ratios >80%). As for *Phytophthora*, fluopimomide exhibited similar efficiency (79.56–89.21%) to fluopicolide (84.64–87.59%). As time passed, the inhibition efficiency of fluopimomide remained constant until 60 DAT. Combined with the results of Table 2, we could conclude that the recovery of fungi amount in fluopimomide treatment at 60 DAT has nothing to do with the target pathogens. Moreover, one-off soil treatment of fluopimomide could effectively control soil-borne pathogens (Table 3).

**Table 3:** Effects of fluopimomide on amounts of soil-borne pathogens in tomato field

Treatment	Soil-borne pathogen numbers		
	20 days	40 days	60 days
*F. oxysporum*/ cfu g^−1^)			
Fluopimomide 375	20.50 ± 2.10c	20.00 ± 2.34c	23.75 ± 2.72c
Fluopimomide 750	18.25 ± 2.29c	16.00 ± 1.96c	19.25 ± 1.38c
Fluopimomide 1,500	13.00 ± 2.48c	12.00 ± 2.27c	12.25 ± 1.65c
Fluopicolide 750	85.75 ± 7.63b	87.25 ± 3.17b	88.00 ± 5.77b
Control	102.5 ± 8.04a	108.25 ± 3.40a	120.25 ± 3.54a
*Phytophthora*/(cfu g^−1^)			
Fluopimomide 375	14.00 ± 1.87b	14.50 ± 1.04b	14.50 ± 0.96b
Fluopimomide 750	11.00 ± 1.58b	11.00 ± 1.08b	11.75 ± 1.18bc
Fluopimomide 1,500	10.50 ± 1.71b	8.50 ± 1.04b	8.25 ± 1.11c
Fluopicolide 750	8.50 ± 0.64b	10.75 ± 2.75b	11.75 ± 0.85bc
Control	68.50 ± 4.05a	73.25 ± 4.25a	76.50 ± 3.43a
*B. cinerea*/(cfu g^−1^)			
Fluopimomide 375	13.25 ± 2.06c	12.25 ± 3.30c	12.75 ± 2.56c
Fluopimomide 750	10.00 ± 1.47c	15.00 ± 2.74c	11.25 ± 1.93c
Fluopimomide 1,500	8.50 ± 1.85c	11.25 ± 2.81c	12.00 ± 2.58c
Fluopicolide 750	40.5 ± 2.60b	46.25 ± 3.01b	46.25 ± 2.43b
Control	77.75 ± 5.16a	86.25 ± 3.50a	89.25 ± 4.13a

The results showed as mean value ± std. error, different letters in each column showed the significant difference at 0.05 level, and the same as follows.

**Table 4:** Control efficiency of fluopimomide on tomato seedling diseases

Treatment	Disease incidences/%	Disease index	Inhibition ratios/%
**Wilt**			
Fluopimomide 375	5.50 ± 0.25c	3.00 ± 0.58c	80.00
Fluopimomide 750	0.00 ± 0.00d	0.00 ± 0.00d	100
Fluopimomide 1,500	0.00 ± 0.00d	0.00 ± 0.00d	100
Fluopicolide 750	22.50 ± 0.25b	18.00 ± 0.82b	18.18
Control	27.5 ± 0.48a	29.00 ± 3.51a	—
**Blight**			
Fluopimomide 375	5.00 ± 0.29b	4.00 ± 0.58b	88.24
Fluopimomide 750	0.00 ± 0.00d	0.00 ± 0.00d	100
Fluopimomide 1,500	0.00 ± 0.00d	0.00 ± 0.00d	100
Fluopicolide 750	2.50 ± 0.25c	2.00 ± 0.5c	94.12
Control	42.50 ± 0.48a	36.50 ± 0.91a	—
**Gray mold**			
Fluopimomide 375	5.00 ± 0.29c	1.50 ± 0.96c	84.63
Fluopimomide 750	0.00 ± 0.00d	0.00 ± 0.00d	100
Fluopimomide 1,500	0.00 ± 0.00d	0.00 ± 0.00d	100
Fluopicolide 750	22.5 ± 0.25b	14.50 ± 2.50b	30.77
Control	32.50 ± 0.48a	34.00 ± 3.16a	—

The results showed as mean value ± std. error, different letters in each column showed the significant difference at 0.05 level, and the same as follows.

### 3.3 Control efficiency of fungicide fluopimomide on tomato seedling diseases

The fluorine atom has four effects: analog, electronic, hindering, and penetration, C–F bond with much higher energy than the C–H, and significantly increases the stability and physiological activity of organic fluorine compounds. In this study, the added four fluorine atoms and a methoxy group in fluopimomide have expanded its fungicidal range. This is consistent with the report of Ji et al., in which fluopimomide was revealed to have excellent efficiency on tomato gray mold [25].

Mulugeta et al. have reported that phosphite could protect tomato against blight but were not effectively under higher disease pressure [26]. In this study, with recommended application dosage, fungicide fluopimomide could significantly reduce the seedling disease incidences of tomato, with inhibition ratios of 80.00, 88.24, and 84.63% for wilt, blight, and gray mold, respectively. Still, when the concentration of fluopimomide doubles or quadruples, the infection of above soil-borne pathogens can be definitely inhibited. In contrast with the novel broad-spectrum fungicide, control fungicide fluopicolide can only inhibit the disease incidence of blight, with the inhibition ratio of 94.12% at the recommended application dosage. The results confirmed the previous indoor toxicity tests and showed that fluopimomide could be recommended as an excellent fungicide for tomato disease management (Table 4).

### 3.4 Effect of fluopimomide on soil enzyme activities

#### 3.4.1 Effect of fluopimomide on soil dehydrogenase activities

Dehydrogenase, representative of soil organism metabolism [27], can transfer hydride groups from a substrate to an acceptor such as NAD^+^. It plays an important role in the organic decomposition process, particularly for bacteria, which are the main ultimate consumers and metabolizers of aromatic compounds, such as pesticides [28,29]. As shown in Table 5, at 20 DAT, recommended dosage of fluopimomide significantly increased soil dehydrogenase activities (*p* < 0.05), while quadruple recommended dosage exhibited a significant inhibition effect. At 40 days after treatment, the dehydrogenase activities of soil treated with a quadruple recommended dosage of fluopimomide increased drastically. This may be because of the soil ecosystem alteration with the increasing fungicide concentration, and microorganisms can increase their metabolic activity in response to xenobiotics in the soil [30]. This is in agreement with Monkiedje et al. [31], who had reported a significant inhibition effect of fungicides mefenoxam and metalaxyl on soil dehydrogenase activity. However, Tejada et al. [32] had found a non-significant increase in dehydrogenase activity in pesticide polluted soils. According to Bending et al. [33], the variant responses of soil dehydrogenase activity to fungicides input are determined by soil type and other factors, such as microbial community structure and types of fungicide. At the end of the incubation (60 DAT), the dehydrogenase activities of treated soil recovered to be similar to control, indicating high safety of tested fungicide on soil dehydrogenase.

**Table 5:** Effects of fluopimomide on soil dehydrogenase activities (μg g^−1^)

Treatment	Treatment time (DAT)		
	20	40	60
Fluopimomide 375	20.778 ± 1.515a	16.164 ± 1.335b	15.799 ± 0.803a
Fluopimomide 750	15.052 ± 1.450b	18.719 ± 1.891ab	16.372 ± 1.148a
Fluopimomide 1,500	11.549 ± 0.727c	20.935 ± 2.342a	16.816 ± 2.500a
Fluopicolide 750	17.815 ± 2.529ab	17.050 ± 0.642b	16.590 ± 0.268a
Control	16.207 ± 1.483b	15.530 ± 0.667b	15.764 ± 1.007a

The results showed as mean value ± std. error, different letters in each column showed the significant difference at 0.05 level, and the same as follows.

#### 3.4.2 Effects of fluopimomide on soil phosphatase activities

As shown in Table 6, fluopimomide exhibited a significant activation effect on soil phosphatase activities with recommended dosage at 20 DAT. However, when treated with a quadruple dosage, the soil phosphatase activity decreased significantly compared with the control. Still, the inhibition effect of higher fungicide concentrations continued until 40 DAT. On 60 DAT, the influences of fluopimomide and fluopicolide in various dosages were reduced to be non-significant.

**Table 6:** Effects of fluopimomide on soil phosphatase activities (mg g^−1^)

Treatment	Treatment time (DAT)		
	20	40	60
Fluopimomide 375	0.664 ± 0.037a	0.618 ± 0.022a	0.594 ± 0.039a
Fluopimomide 750	0.503 ± 0.048bc	0.649 ± 0.040a	0.590 ± 0.018a
Fluopimomide 1,500	0.421 ± 0.083c	0.534 ± 0.033b	0.579 ± 0.020a
Fluopicolide 750	0.540 ± 0.015b	0.627 ± 0.026a	0.578 ± 0.029a
CK	0.564 ± 0.029b	0.580 ± 0.029ab	0.583 ± 0.024a

The results showed as mean value ± std. error, different letters in each column showed the significant difference at 0.05 level, and the same as follows.

Our result is in accordance with that of Jastrzębska et al. [34], which reported an increase in soil phosphatase activity after being treated with cyprodinil, dimoxystrobin, and epoxiconazole, and indicated the direct relationship between the increase in soil phosphatase activity and dosage of the applied fungicide. Monkiedje and Spiteller [35] also found the stimulating effects of metalaxyl and prochloraz on soil phosphatase activity and speculated that both fungicides were used as sources of energy by soil microorganisms. Nevertheless, Chen et al. [36] reported the inhibitory effect of benomyl, captan, and chlorothalonil on soil phosphatase activity. In this assay, fluopimomide has exhibited a gentle and restorable effect on soil phosphatase activities.

#### 3.4.3 Effects of fluopimomide on soil urease activities

As reported, urease is externalized due to parent cell death and lysis. This enzyme plays an important role in the nitrogen cycle in soils. Its substrate, urea, is incorporated into the soil from fertilizer, animal excreta, or nucleic acids [17]. In this study, with recommended dosages of fluopimomide and fluopicolide, a non-significant increase has been detected. Still, large dosages of fluopimomide could inhibit urease activity to a certain extent (<10%). With passage of time, the effects of fungicides on soil urease activities became lighter and lighter. In previous studies, similar results have been reported. Monkiedje et al. [35] observed a slight inhibitory effect of metalaxyl and prochloraz on urease in the short term. It was speculated that soil microorganisms took fungicides as an energy source. Uyanőz et al. [37] also reported the activation effect of captan, quintozene, and propamocarb hydrochloride on soil urease activity (Table 7).

**Table 7:** Effects of fluopimomide on soil urease activities (mg 100 g^−1^)

Treatment	Treatment time (DAT)		
	20	40	60
Fluopimomide 375	0.495 ± 0.016a	0.512 ± 0.034b	0.494 ± 0.033a
Fluopimomide 750	0.412 ± 0.041a	0.550 ± 0.050ab	0.503 ± 0.015a
Fluopimomide 1,500	0.336 ± 0.059b	0.631 ± 0.067a	0.522 ± 0.047a
Fluopicolide 750	0.486 ± 0.023a	0.506 ± 0.016b	0.491 ± 0.030a
CK	0.459 ± 0.029a	0.483 ± 0.048b	0.486 ± 0.043a

The results showed as mean value ± std. error, different letters in each column showed the significant difference at 0.05 level, and the same as follows.

#### 3.4.4 Effects of fluopimomide on soil invertase activities

Soil invertase is of particular importance in carbon cycles [38]. A previous study had documented that bioorganic fertilizer application could always improve invertase activity [39]. Our assay results showed a significant increase in invertase activities when the soil was treated with a recommended dosage of fluopimomide and fluopicolide at 20 DAT, which were probably used as carbon sources by microbes. However, the activation effects were converted to inhibition at 40 DAT, and recovered to be similar to control at 60 DAT. The complicated influence of fluopimomide may be due to the complex response of microorganisms to fungicide dosages, including the ecology change in the microorganism community.

Overall, the effects of fluopimomide on the four soil enzymes are temporary and reversible, indicating relatively high safety to the soil environment (Table 8).

**Table 8:** Effects of fluopimomide on soil invertase activities (mg 100 g^−1^)

Treatment	Treatment time (DAT)		
	20	40	60
Fluopimomide 375	19.086 ± 1.690a	14.684 ± 1.150c	16.331 ± 0.889a
Fluopimomide 750	20.059 ± 1.865a	17.413 ± 1.804ab	14.076 ± 1.511a
Fluopimomide 1,500	17.420 ± 0.910ab	19.408 ± 1.849a	14.971 ± 3.824a
Fluopicolide 750	19.247 ± 1.048a	13.281 ± 1.078c	15.197 ± 2.714a
CK	15.124 ± 1.802b	16.122 ± 1.358b	15.441 ± 2.166a

The results showed as mean value ± std. error, different letters in each column showed the significant difference at 0.05 level, and the same as follows.

### 3.5 Quantitative PCR analysis of soil nitrogen-related genes

**Figure 2:**
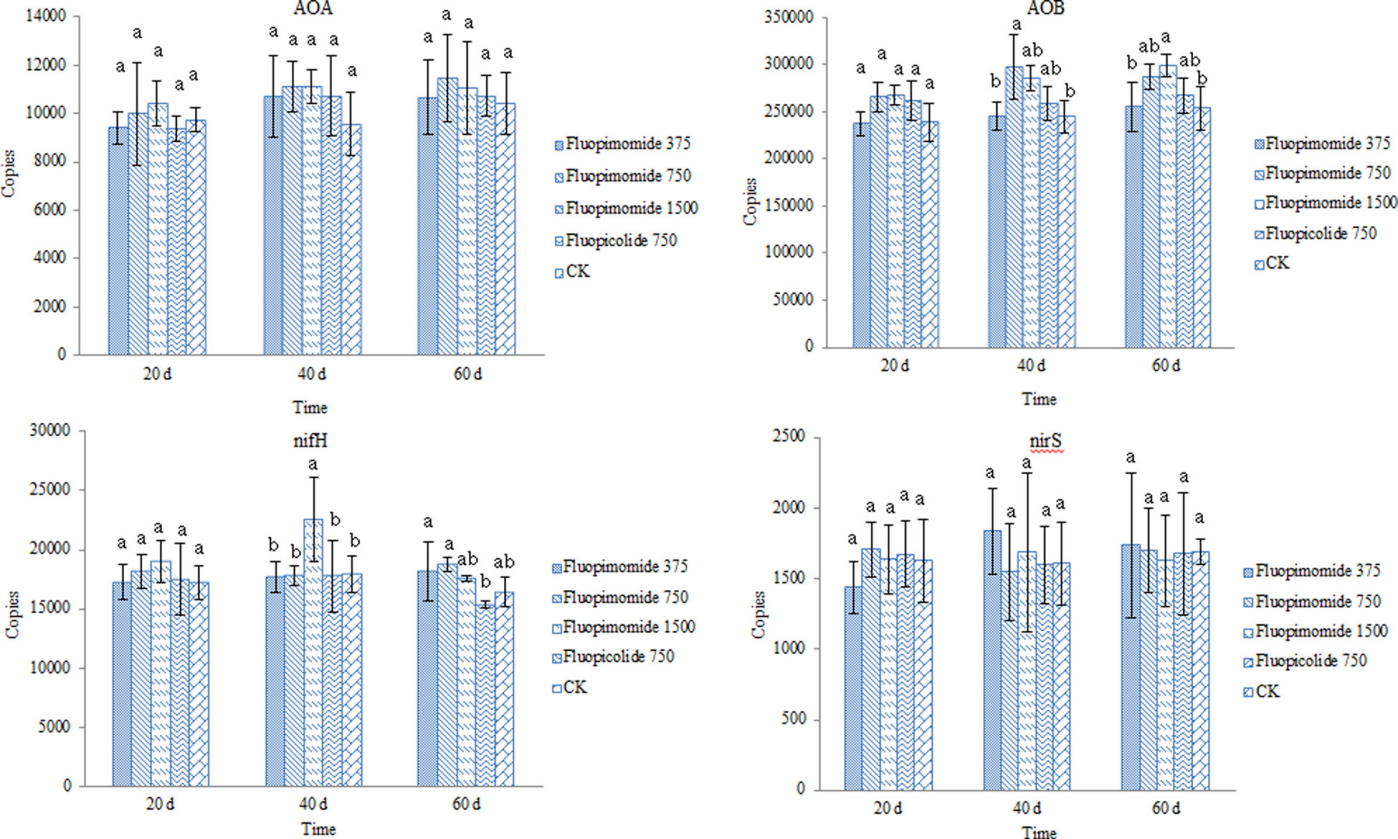
Effects of fluopimomide on soil nitrogen-related genes.

Soil microbes are essential in soil nutrient mineralization and accumulation [40]. Soil N cycling is participated by varieties of microorganisms, of which *nifH* encodes for N_2_ fixation, AOA and AOB for ammonia oxidation, and *nirS* for denitrification [41]. Jiang et al. has reported that five fluoroalkylether compounds could reduce *amoA* gene abundance in soil and had different effects on *nirS* [42]. In this study, 20, 40, and 60 DAT, the copy numbers of AOA and *nirS* fluctuated greatly, but the differences among treatments were not obvious, indicating that the effects of chemical treatment on AOA and denitrifying bacteria in soil were not regular. At 40 and 60 DAT, the AOB and *nifH* gene copy numbers were higher in the double and quadruple dosages of fluopimomide treatments than in the control, indicating that fluopimomide could promote the proliferation of soil AOB, nitrogen-fixing bacteria, and other bacteria (Figure 2), which might be helpful for tomato plant growth [43].

### 3.6 Effect of fluopimomide on plant height and marketable yield

Soil management for disease suppressive could help to improve plant growth [44]. Jakl et al. has reported the increased effect of soil-treated triazole fungicides on tomato fruit yield [45]. As shown in Table 9, 20 DAT, fluopimomide had increased tomato plant height non-significantly by 13.25, 25.65, and 20.03% with 1-, 2-, and 4-folds of recommended dosages. Still, the stimulated efficiencies continued with time-lapse until 60 DAT. A similar tendency could be found in fluopicolide as well. Meanwhile, the two fungicides significantly improved marketable tomato yield by reducing disease incidences. Among which fluopimomide had improved the total marketable yield by 16.88, 18.16, and 17.47%, with the application dosage of 375, 750, and 1,500 g ha^−1^, respectively. Furthermore, fluopicolide exhibited lower stimulation efficiency by 9.87%. Thus, the conclusion could be drawn out that fungicide fluopimomide could improve tomato yield, possibly via the inhibition of soil-borne pathogens.

**Table 9:** Effects of fluopimomide on tomato plant height and marketable yield

Fungicide	Plant height/cm			Marketable yield (t/ha)
	20 DAT	40 DAT	60 DAT	
Fluopimomide 375	14.70 ± 0.90ab	42.86 ± 2.90ab	75.73 ± 2.07ab	59.20 ± 1.28a
Fluopimomide 750	16.31 ± 0.69a	44.22 ± 2.62ab	78.20 ± 4.38a	59.85 ± 1.11a
Fluopimomide 1,500	15.58 ± 0.87ab	43.53 ± 2.17ab	76.78 ± 4.21a	59.50 ± 1.65ab
Fluopicolide 750	15.06 ± 0.87ab	43.26 ± 3.31ab	72.88 ± 2.72ab	55.65 ± 1.03b
Control	12.98 ± 0.34b	34.55 ± 1.24b	66.50 ± 0.81b	50.65 ± 0.57c

The results showed as mean value ± std. error, different letters in each column showed the significant difference at 0.05 level.

## 4 Conclusion

Via soil treatment, the new fluorinated benzamide fungicide fluopimomide could significantly reduce the amounts of soil-borne pathogens in soil, reduce the disease incidences in tomato plants, and eventually increase the marketable yield of tomatoes. During the inoculation period, the soil enzymes had been influenced differently, and AOB and *nifH* gene copy numbers were increased by the double and quadruple dosages of fluopimomide treatment. Compared with the control fungicide fluopicolide, fluopimomide exhibited more efficiency in tomato plant height and marketable yield. Therefore, as a broad-spectrum fungicide, fluopimomide could be popularized to manage tomato diseases. Still, the broader application scope remains to be investigated.
